# Recent advances in understanding the role of IL-4 signaling

**DOI:** 10.12703/r/10-71

**Published:** 2021-09-07

**Authors:** Achsah D Keegan, Warren J Leonard, Jinfang Zhu

**Affiliations:** 1 Center for Vascular and Inflammatory Diseases, Department of Microbiology and Immunology, University of Maryland School of Medicine, and Veterans Affairs Maryland Health Care System, Baltimore Veterans Affairs Medical Center, Baltimore, USA; 2 Laboratory of Molecular Immunology, Immunology Center, National Heart, Lung, and Blood Institute, National Institutes of Health, Bethesda, USA; 3 Molecular and Cellular Immunoregulation Section, Laboratory of Immune System Biology, National Institute of Allergy and Infectious Diseases, National Institutes of Health, Bethesda, USA

**Keywords:** IL-4, IL-13, cytokine, STAT6, receptor, IL-4Ralpha1, IL-13Ralpha1, common gamma chain

## Abstract

Interleukin-4 (IL-4) is a four-α-helical bundle type I cytokine with broad pleiotropic actions on multiple lineages. Major actions of IL-4 were initially discovered for B and T cells, but this cytokine acts on more than a dozen different target cells spanning the innate and adaptive immune systems and is produced by multiple different cellular sources. While IL-4 was discovered just under 40 years ago in 1982, the interest in and discoveries related to this cytokine continue to markedly expand. There are important new advances related to its biological actions and to its mechanisms of signaling, including critical genes and downstream targets in a range of cell types. IL-4 is critical not only for careful control of immunoglobulin production but also related to inflammation, fibrosis, allergic reactions, and antitumor activity, with actions of IL-4 occurring through two different types of receptors, one of which is also used by IL-13, a closely related cytokine with partially overlapping actions. In this review, we cover critical older information but also highlight newer advances. An area of evolving interest relates to the therapeutic blockade of IL-4 signaling pathway to treat atopic dermatitis and asthma. Thus, this cytokine is historically important, and research in this area has both elucidated major biological pathways and led to therapeutic advances for diseases that affect millions of individuals.

## Overview

Interleukin 4 (IL-4) was first identified as a factor that was produced by T cells and could enhance the proliferative response of B cells to anti-IgM and also was capable of inducible immunoglobulin (Ig) isotype switch of B cells to produce IgG, particularly IgG1^1,2^. Subsequently, this cytokine was demonstrated to be even more pleiotropic/polyfunctional. For example, IL-4 is also the key cytokine for the differentiation of T helper 2 (Th2) cells, which control infection by extracellular parasites and contribute to allergic responses^3,4^, and it can induce the differentiation of M2 macrophages, which control infection by the protozoan *Trypanosoma cruzi*^5^. IL-4 acts through two types of receptors—the type I and type II IL-4 receptor—which share the IL-4 receptor α chain but have a different secondary receptor, γ_c_ versus IL-13Rα1, respectively (discussed in detail in the text), in part depending on cell type, and a highly related cytokine, IL-13, uses only the type II IL-4 receptor. Not only have there been extraordinary advances in the biology of these cytokines and their signaling mechanisms, but because of the critical roles of IL-4 related to the mediation of allergic responses, controlling the actions of this cytokine has been of profound therapeutic interest. Indeed, we now are at a point of tremendous excitement where blockade of IL-4 and IL-13 is effective in the treatment of allergic inflammatory responses, including atopic dermatitis and moderate to severe asthma.

## Biology of IL-4

**Figure 1.**
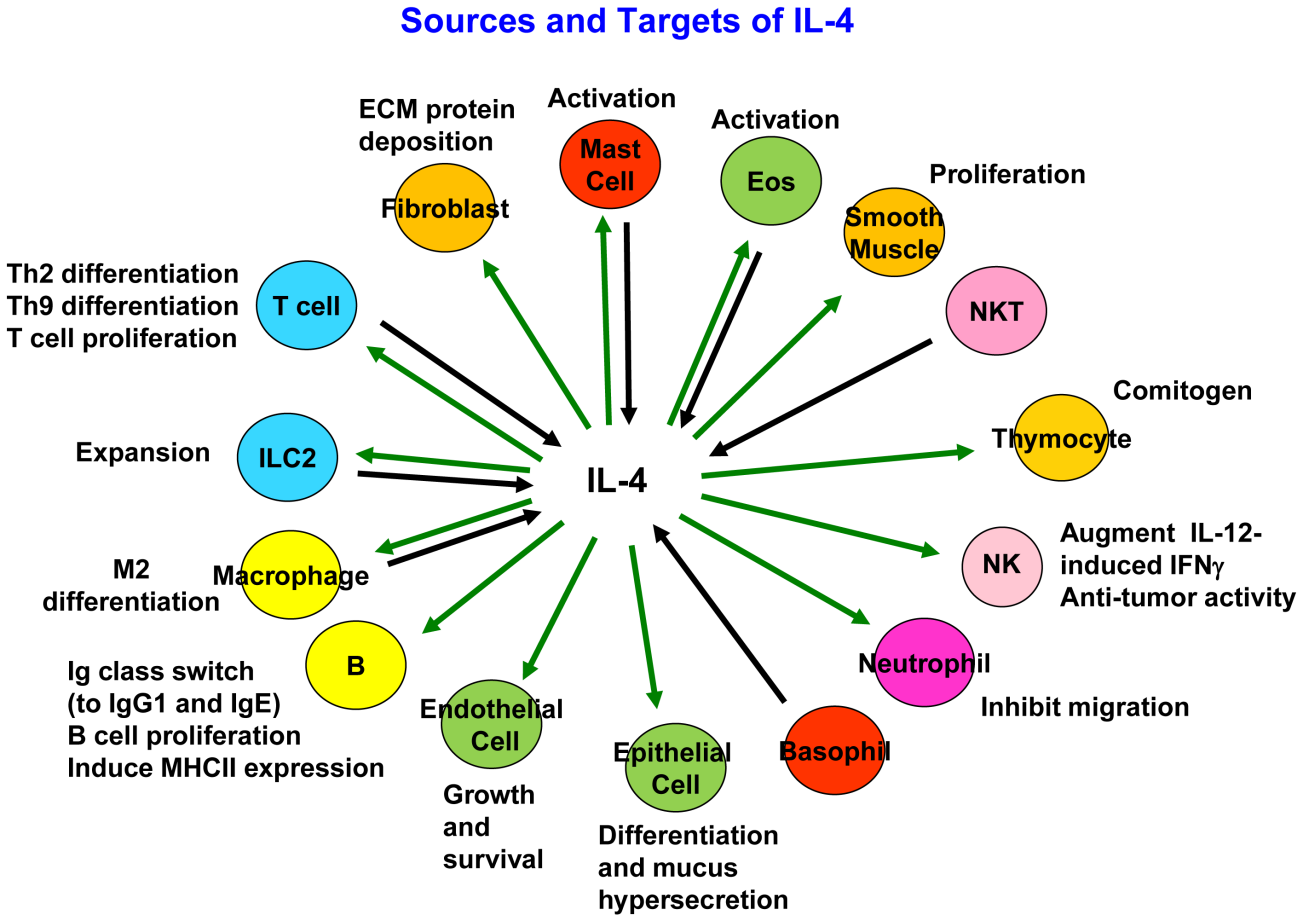
Schematic of cellular sources of interleukin 4 (IL-4) and target cells for IL-4. IL-4 potentially acts on other cells that are not listed. ECM, extracellular matrix; IFNγ, interferon gamma; MHC, major histocompatibility complex; NK, natural killer; NKT, natural killer T; Th, T helper.

Although IL-4 is produced primarily by activated CD4^+^ T cells^4^, it also is produced by CD4^+^NK1.1^+^ “natural” T (NKT) cells, macrophages, eosinophils, basophils, mast cells, and type 2 innate lymphoid cells (ILC2s), although the amounts of IL-4 produced by these cell types are not uniform^6–8^ (Figure 1). IL-4 was initially described as a major B-cell growth factor and a promoter of Ig class switch that enhanced the production and secretion of mouse IgG1 (human IgG4)^1,2^. Subsequent work revealed that IL-4 is essential for the production of IgE, which is critical for allergen sensitization as well as the physiological response to parasites, including helminths. IL-4 induces elevated cell surface expression of CD23 (the low-affinity IgE receptor) on B cells and of class II major histocompatibility complex (MHC) molecules. In addition, IL-4 acts as a T-cell growth factor in both humans and mice and promotes the differentiation of Th2 cells. When combined with phorbol 2-myristate 3-acetate, IL-4 is also a potent co-mitogen for thymocytes. Furthermore, IL-4 derived from NKT cells is responsible for the expansion of memory-like CD8 T cells in BALB/c mice^9^. Overall, IL-4 can exert actions on a broad range of target cells, including macrophages, hematopoietic precursor cells, stromal cells, NK cells, and fibroblasts^7^, and can act as a potent anti-apoptotic factor.

## IL-4 receptors

**Figure 2.**
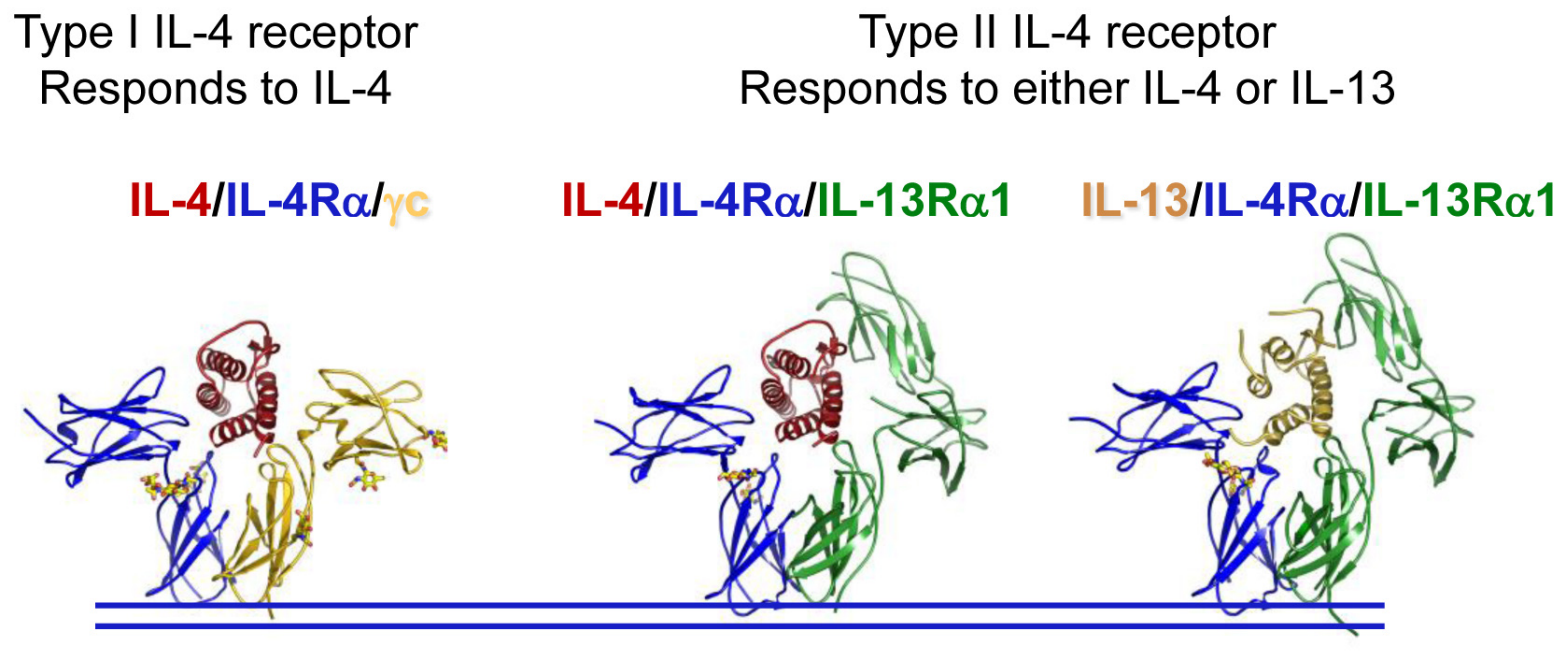
Crystal structure of the type I interleukin 4 (IL-4) receptor bound to IL-4 (left) or the type II IL-4 receptor bound to either IL-4 (center) or IL-13 (right). This figure was adapted using parts of Figure 3 from Elsevier, 132, LaPorte *et al*., Molecular and Structural Basis of Cytokine Receptor Pleiotropy in the Interleukin-4/13 System, 259–72, 2008^14^, with permission from Elsevier.

As noted above, IL-4 acts on both lympho-hematopoietic and non-lympho-hematopoietic cells^10^. Interestingly, there are two types of IL-4 receptors (Figure 2): type I IL-4 receptors are composed of the 140-kDa IL-4Rα and γ_c_ and are expressed on lympho-hematopoietic cells^11,12^; in contrast, type II IL-4 receptors comprising IL-4Rα and IL-13Rα1 are found on non-hematopoietic cells, and type II receptors also serve as the functional receptor for IL-13^13^, and both IL-4 and IL-13 induce similar actions via this type of receptor. Expression of IL-4Rα tends to be quite low, and cells that potently respond to IL-4 often express only a few hundred receptors per cell. For example, there are only about 300 IL-4 binding sites on resting lymphocytes, although receptor numbers can increase 5- to 10-fold upon cellular activation^4^. In reconstitution experiments, it was found that IL-4 binds to IL-4Rα with strong affinity (K_d_ ~ 266 pM), and in the presence of γ_c_, the binding affinity is increased to “high” affinity (K_d_ ~79 pM)^12^, with the interaction of IL-4-IL-4Rα with γ_c_ being weak (K_d_ in > 500 nM)^14^. The type II IL-4 receptor, containing IL-4Rα and IL-13Rα1, is not expressed on mature T cells but is expressed on multiple other cell types and can transduce signals in these cells. Interestingly, type II receptors are expressed on immature T cells, including neonatal Th1 cells, and these receptors are implicated as mediating apoptosis of these cells^15^. The differential expression of γ_c_ and IL-13Rα1 determines whether IL-4 will signal via the type I or type II receptor, and IL-13 shares effects with IL-4 on the cells with type II IL-4 receptors. Consistent with this functional similarity, IL-4 and IL-13 are encoded by adjacent genes on mouse chromosome 11 and closely positioned on human chromosome 5q31; in both species, they are located between the *RAD50* and *KIF3A* genes, consistent with a common ancestral gene. Interestingly, the genes encoding γ_c_** and IL-13Rα1 are both located on the X chromosome and share some general properties, suggesting that they may also have arisen by gene duplication.

Both *Il4*^−/−^ and *Il4ra*^−/−^ mice have normal numbers of T and B cells, indicating that IL-4 and IL-13 are not required for development of these lineages, but they have diminished IgG1 production and greatly decreased IgE production, after infection with *Nippostrongylus brasiliensis*, whereas levels of IgM and other IgG isotypes are produced at normal levels^16,17^. Interestingly, another γ_c_ family cytokine, IL-21, has some properties similar to those of IL-4 but also major differences. For example, the development of T and B cells is normal in *Il21r*^−/−^ mice; however, IgG1 levels are greatly diminished, and IL-21 is also required for IgG3 production by CD40-activated naïve human B cells^18,19^. Whereas IL-4 favors the production of IgG1, IL-21 favors IgG3 production by CD40-activated naïve human B cells^18^, and whereas mice lacking IL-4 signaling have low IgE, IgE levels are elevated in *Il21r*^−/−^ mice after immunization^20^, and this may be at least in part due to an inhibitory effect of IL-21 on IL-4-induced germ line Cε transcription^21^. IL-4 and IL-21 appear to cooperate for Ig production as indicated by a pan-hypogammaglobulinemia and poorly organized germinal centers in mice lacking both IL-4 and IL-21 signaling^20^. According to these observations, both IL-4 and IL-21 contribute to Ig production, Ig isotype switch, and the differentiation of B cells into plasma cells. Interestingly, cells that express only IL-4 are more efficient at promoting IgG1 class switch and plasma cell differentiation, whereas cells that secrete only IL-21 are more efficient at promoting somatic hypermutation and affinity maturation in B cells, and cells that express both IL-4 and IL-21 can serve both functions^22^. This illustrates the important but complex manner in which IL-4 and IL-21 functionally interact in B-cell biology.

## IL-4 signaling

**Figure 3.**
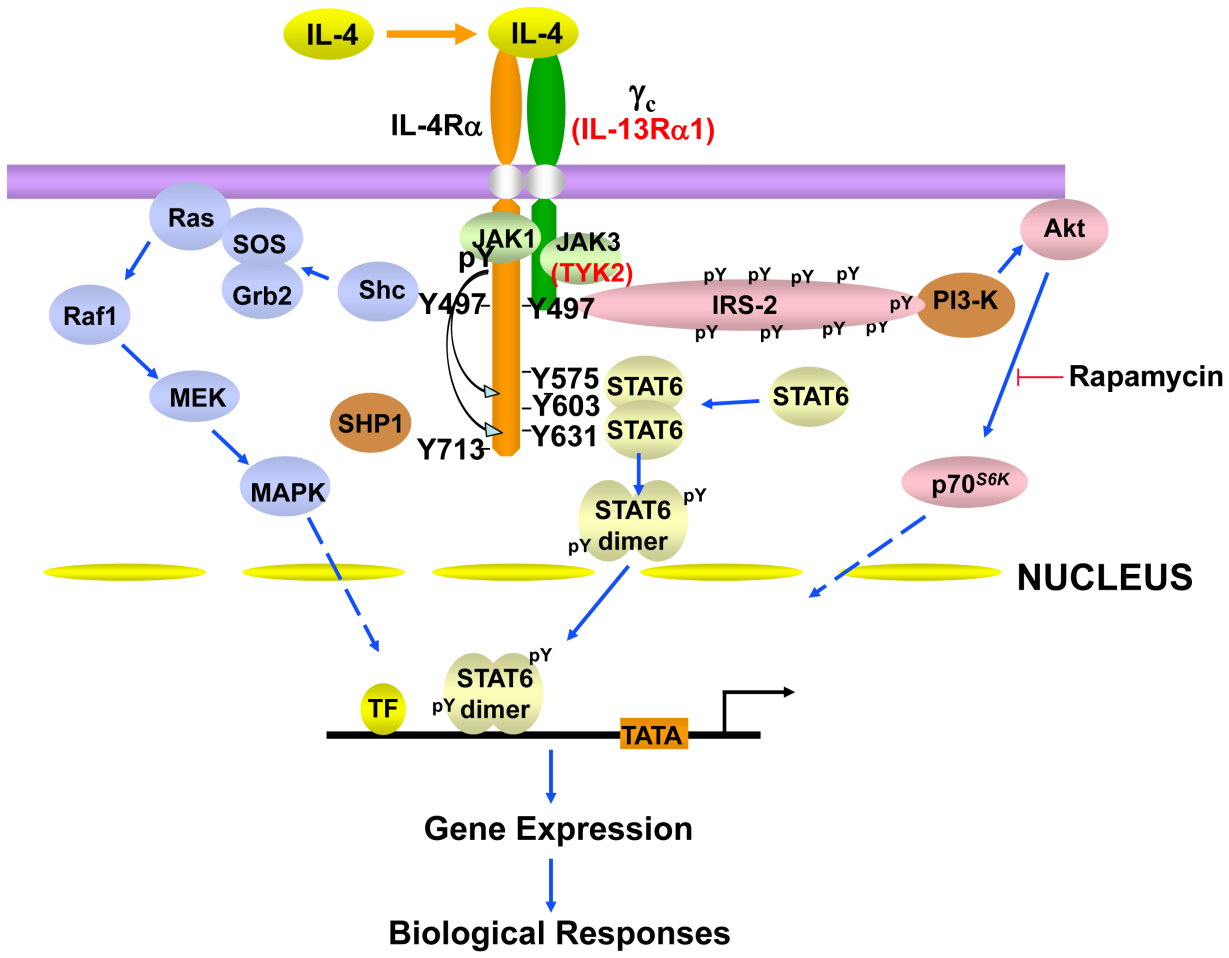
Schematic of some of the major signaling pathways and molecules involved in interleukin 4 (IL-4) signaling. IL-4 signaling via the type I IL-4 receptor, consisting of IL-4Rα and γ_c_, activates JAK1 and JAK3, whereas JAK1 and TYK2 are the tyrosine kinases involved in IL-4 signaling via the type II IL-4 receptor, which consists of IL-4Rα and IL-13Rα1. Type II IL-4 receptor–specific molecules are shown in red font. IRS2 and Shc interact with phosphorylated Y497, whereas Y575, Y603, and Y631, when phosphorylated, are docking sites for the SH2 domain of STAT6. Collectively, these signaling molecules mediate IL-4 signaling as well. IL-13 signals via the type II IL-4 receptor and activates JAK1 and either JAK2 or TYK2 but not JAK3. Nevertheless, the major signaling pathways are the same as what is shown given that IL-4Rα is the dominant molecule for the docking of signaling proteins. All of the positions of tyrosines indicated in the figure are derived from human IL-4Rα. STAT3 can also be recruited to the IL-13Rα1 in the type II receptor in human macrophages.

Like the other γ_c_ family cytokines, IL-4 activates multiple signaling pathways (Figure 3). IL-4 activates JAK1 and JAK3 via the type I IL-4 receptor; however, IL-4 activates JAK1 and either JAK2 or TYK2 (depending on the cell type) via type II IL-4 receptors. Although there are subtle signaling differences between the type I and type II receptors^23^, IL-4 and IL-13 are both distinctive in their potent activation of STAT6, which docks on key phosphotyrosines on IL-4Rα (Figure 3)^24,25^. STAT6 is so vital for the actions of IL-4 that *Stat6*-deficient mice phenocopy many of the defects observed with *Il4*- or *Il4ra*-deficient mice (e.g., related to Th2 differentiation and Ig class switch). However, in addition to JAK-STAT activation, like insulin, IL-4 can induce tyrosine phosphorylation of an IRS1-like molecule in hematopoietic cells, denoted IRS2^26,27^. Studies in *Irs2*-deficient mice indicate that IL-4-activated IRS2 mediates negative regulatory feedback for the PI3K pathway in macrophages by targeting the phosphorylation of IRS1.

Recently, there has been considerable interest in IL4I1, which is encoded by an early IL-4-inducible gene in B cells^28^ that belongs to the L-amino-acid oxidase family and catalyzes the oxidation of amino acids, including L-phenylanine^29^. It inhibits T-cell proliferation by releasing its enzymatic catabolite, hydrogen peroxide, which results in lower expression of T-cell receptor zeta (TCRζ). However, it also contributes to macrophage programming and is a regulator of M2 macrophage polarization that inhibits T-cell activation via L-tryptophan and L-arginine depletion as well as increased IL-10 production^30^. Moreover, it activates the aryl hydrocarbon receptor and promotes tumor progression^31^. IL4I1 also was reported to be a prognostic biomarker affecting the local T-cell response in human cutaneous melanoma^32^ and to be capable of promoting central nervous system remyelination by modulating T cell–driven inflammation^33^.

## Effects of IL-4 on T-cell differentiation

IL-4 is one of the key signature cytokines of Th2 cells^3,34–37^. Interestingly, IL-4 can also drive Th2 cell differentiation, which constitutes a powerful positive feedback mechanism during T-cell differentiation^38^. IL-4 may directly act on naïve CD4 T cells during T-cell activation to induce Th2 cell differentiation. STAT6 activation by IL-4 is necessary and sufficient during this process^39^. The initial source of IL-4 could be basophils, NKT cells, or naïve CD4 T cells themselves. Low dose of antigen stimulation may result in IL-2-dependent STAT5-driven early IL-4 production, which in turn further promotes Th2 cell differentiation^40^. Both IL-2- and IL-4-mediated signaling are also capable of inducing the expression of IL-4Rα, which can further enhance cellular responsiveness to IL-4^39,41,42^. IL-4/STAT6 signaling in activated CD4 T cells induces the expression of the Th2 master transcription factor GATA3, which is critical for Th2 cell function and maintenance^43,44^. GATA3 directly activates *Il5* transcription^45^; it also plays an important role in chromatin remodeling at the *Il4/Il13* cytokine locus during Th2 cell differentiation^46–48^. In addition, the activation of STAT5 proteins (activated by IL-2 and potentially by other cytokines) is essential for initiating and maintaining Th2 differentiation^44,49,50^. IL-2 induces IL-4Rα expression early in Th2 differentiation, facilitating responsiveness of cells to IL-4, which promotes and sustains GATA3 expression. The induction of IL-4Rα by IL-2 is attributed to the binding of STAT5 proteins to the GAS motifs in the first intron of the *Il4ra* gene, and constitutive expression of *Il4ra* in *Il2*^−/−^ CD4^+^ T cells restores Th2 differentiation in these cells^51^. Many Th2-specific genes, including those encoding IL-5, IL-13, and T1/ST2 (IL-33 receptor α chain), are GATA3 direct targets^46^; the clustering of some of these genes may help to explain their coordinated induction during Th2 differentiation. IL-4 can also expand GATA3-expressing Th2 cells through STAT6-mediated upregulation of transcription factor Gfi-1^52^. IL-4 not only can drive Th2 cell polarization from naïve CD4 T cells but also induces a Th2 cell phenotype in already-differentiated Th cells, including Th17 cells^53^. Genome-wide CRISPR screens have also been performed and have revealed crosstalk between differentiation and activation in the Th2 differentiation process, underscoring the roles of factors, including *Pparg* and *Bhlhe40*, in controlling Th2 cell differentiation^54^.

IL-4 may also indirectly promote Th2 cell differentiation. It has been reported that IL-4 may induce TSLP^55^, which is a critical mediator of Th2 responses *in vivo* through its actions on dendritic cells (DCs) or T cells^56–59^. IL-4 and IL-13 can also act directly on intestinal epithelium to induce tuft cells to produce IL-25^60^, a potent activator of ILC2s, which are the counterparts of Th2 cells within the innate immune system^8,61–63^. ILC2s can mediate type 2 inflammation through their production of IL-5 and IL-13 even in the absence of Th2 cells, but under certain circumstances, ILC2s may also promote the differentiation of CD4^+^ T cells toward the Th2 cell fate^64^. Furthermore, ILC2s and Th2 cells may directly interact with each other through MHCII–TCR interaction^65^. Therefore, ILC2s and Th2 cells may exhibit crosstalk and collaborate in type 2 immune responses *in vivo* via multiple mechanisms^8,61,66–68^.

Although IL-4 seems to be required for Th2 cell differentiation *in vitro*, both IL-4-dependent and IL-4-independent Th2 cell differentiation have been reported *in vivo*^3^. Although GATA3 is absolutely required for Th2 cell differentiation both *in vitro* and *in vivo*, Th2 cell differentiation may occur even in CD4 T cells expressing low levels of GATA3 if these cells also receive a strong STAT5 signal^44,50^. Multiple cytokines, including IL-2, IL-7, IL-9, IL-15, and TSLP, can activate STAT5^69,70^, and TSLP has been shown to program a pathogenic Th2 cell state^71^, which may provide a basis for IL-4-independent but GATA3-dependent Th2 cell differentiation *in vivo*. Indeed, our unpublished data indicate that high amounts of IL-7 or TSLP are capable of inducing Th2 cell differentiation to a certain degree in the absence of IL-4 signaling *in vitro*. This finding may partially explain the difference between *in vitro* and *in vivo* differentiation. However, it is possible that *in vivo* cell–cell interactions through co-stimulatory molecules, such as OX-40^72,73^ or Notch signaling^74^, are involved in IL-4-independent Th2 cell differentiation.

A recent study further assessed the requirement of IL-4 signaling for the differentiation of Th2 cell subsets *in vivo*^75^. Interestingly, in draining lymph nodes, while the development of IL-4-producing T follicular helper (Tfh) cells is not affected, non-Tfh IL-4-producing cells (Th2 effector cells) are reduced in the absence of IL-4 signaling. Furthermore, in dermal and lung tissues, whereas the generation of IL-13-only-producing Th2 cells is IL-4-independent, the generation of IL-4-producing Th2 cells (with or without IL-13 co-expression) depends on IL-4 signaling. Interestingly, TSLP drives the differentiation of IL-13-expressing Th2 cells^76^. These results indicate that IL-4 and TSLP can influence the differentiation or the expansion (or both) of distinct Th2 cell subsets. IL-4 can act in an autocrine fashion related to cell proliferation and survival, which may explain the preferential expansion of IL-4-producing Th2 cells as compared with other Th2 cells *in vivo*.

Together with transforming growth factor beta (TGFβ), IL-4 through activating STAT6 has been reported to induce IL-9-producing (Th9) cells from either naïve CD4 T cells or from Th2 cells^77–79^. Not only can IL-4-producing Th2 cells convert into IL-9-producing Th9 cells, IL-9-producing cells can—according to our unpublished data—also become IL-4-producing cells in the absence of TGFβ. Therefore, it is possible that a transcription factor whose expression is regulated by the balance between TGFβ and IL-4 serves as a switch for determining conversion from Th2 to Th9 cells or vice versa. Interestingly, together with IL-1β, IL-4 can induce the differentiation of anti-tumor Th9 cells in the absence of TGFβ signaling^80^, and these cells are phenotypically distinct from the Th9 cells induced by the combination of IL-4 and TGFβ.

## IL-4 on ILC2s and other ILCs

Like Th2 cells, ILC2s can produce IL-5 and IL-13; although ILC2s also produce IL-4 under certain circumstances^81–84^, they produce much less IL-4 than Th2 cells^85^. However, the ability of ILC2s to produce IL-4 *in vivo* and the physiological importance of such production require further investigation. Nevertheless, ILC2s can be identified by an *Il4* reporter construct in mice in steady state and after IL-25 treatment, indicating that the *Il4* locus has an open chromatin configuration in these cells^86^. The reason why ILC2s produce much less IL-4 than Th2 cells do is unknown, but it seems likely that the chromatin structure at the Th2 cytokine loci containing *Il4/Il13/Il5* is quite distinct^87^. Interestingly, lung Th2 cells produce more IL-13, but draining lymph node Th2 cells produce more IL-4^88,89^; thus, it is also possible that preferential expression of IL-13 over IL-4 or vice versa is determined in a tissue-specific fashion^90^. GATA3 expression levels vary between ILC2s and Th2 cells in different locations. Thus, it is conceivable that the level of expression of GATA3, by regulating the formation of distinct chromatin structures at the Th2 cytokine loci, determines expression of IL-4 versus IL-13. Although many studies have demonstrated the physiological importance of IL-13 production by ILC2s, fewer have studied the importance of IL-4 production by these cells. Interestingly, however, it was reported that IL-4 produced by ILC2s drives Th2 cell differentiation^84^ and that IL-4 produced by ILC2s in IL-4Rα Y709F mutant mice (Y709 in mice corresponds to Y713 in humans) may promote food allergy by blocking the generation of allergen-specific regulatory T cells^91^.

ILC2s can be divided into IL-33-responsive nILC2s (natural ILC2s) and IL-25-responsive iILC2s (inflammatory ILC2s)^92,93^. iILC2s are located mainly in the gut but can migrate to the lung tissue after *Nippostrongylus brasiliensis* infection^94^ or IL-25 treatment^95^ and produce both IL-4 and IL-13^94^. Interestingly, deficiency of BATF, a Fos family member that is a component of AP1 complexes^96^ and that helps to form AP1-IRF4 composite elements^97–100^, selectively affects iILC2s but not nILC2s in the lung after infection^94^. iILC2s may turn into nILC2-like cells or ILC3-like cells^92^. c-Kit-expressing ILC2s in humans may resemble iILC2s in mice, and these cells can be converted into RORγt-expressing cells by IL-1 and IL-23 stimulation; this conversion is promoted by TGFβ but inhibited by IL-4^101^. By contrast, during ILC2 development, TGFβ was recently reported to induce T1/ST2 expression and thus to promote ILC2 development from its progenitors^102^. IL-1 together with IL-12 may convert ILC2s into interferon gamma (IFNγ)-producing ILCs, but IL-4 can inhibit this effect^103–105^. In addition to an important role of IL-4 in maintaining ILC2 identity, IL-4 or IL-13 (or both) made by Th2 cells may act directly on ILC2s to induce local expansion of ILC2s during type 2 immune responses^106^. Furthermore, basophil-derived IL-4 controls ILC2 actions, including the secretion of IL-13 during protease allergen–induced airway inflammation^107^.

## IL-4 and macrophages

IL-4 is known to regulate the phenotypes of macrophages^108–116^. Since macrophages are mediators of host defense and play critical roles in a range of physiological processes, including tissue homeostasis and repair, recent studies have focused on understanding macrophage populations that mediate these specific functions in the context of diverse settings such as allergic inflammation, nematode infection, cancer, muscle regeneration, and thermogenesis^117–125^. Macrophages can adopt a wide spectrum of phenotypes controlled by tissue and environmental signals. A pro-inflammatory phenotype exists at one end of the spectrum (previously termed “M1” for lipopolysaccharide [LPS]/IFNγ-activated macrophages), and the pro-tissue repair phenotype at the other end (previously termed “M2,” “M2a,” or alternatively activated macrophages [AAMs] for the IL-4- or IL-13-activated macrophages). These strongly polarized phenotypes were initially characterized *in vitro* where addition of specific recombinant cytokines could push phenotypes to extremes in isolation^108,109^. M1 cells are typically found in abundance in tissues during infections with intracellular pathogens, exposure to components of bacterial cell walls, and high levels of IFNγ. In contrast, M2 cells are found in abundance in tissues high in IL-4 and IL-13, as is seen in parasitic worm infection or allergic inflammation. However, it is now appreciated that the heterogeneity of macrophage phenotypes is more complex *in vivo*, and traditional markers used for M1 versus M2 delineation, CD86 and CD206, respectively, are not sufficient to resolve the complexity of macrophage phenotypes *in vivo*^113,115,116,126^. Furthermore, the limited models of macrophage-specific or -selective deletion make it difficult to unambiguously assign roles for certain types of macrophages in disease processes^127–129^.****

To better phenotype expression of markers by macrophage populations in tissue, Sommerfeld *et al*.^130^ recently used single-cell RNA sequencing (scRNA-Seq) to characterize macrophage phenotypes in mouse muscle tissue undergoing injury response to agents that drive either a pro-fibrotic (F) or a pro-regenerative (R) response. They identified two unique macrophage phenotypes in muscle macrophages in each condition (F1, F2 and R1, R2 respectively). Under fibrotic conditions, muscle macrophages (defined as CD45^+^CD64^+^F4/80^hi^) were characterized by gene expression as CD9^−^CD301b^−^MHCII^hi^ (F1) and CD9^hi^CD301b^−^ MHCII-IL-36g^+^ (F2). Under regenerative conditions, muscle macrophages were characterized as CD9^+^CD301b^+^MHCII^hi^ (R1) and CD9^−^CD301b^+^CD206^+^ (R2). Interestingly, the R2 macrophages were sustained in the tissue for up to 6 weeks after injury and upregulated genes that are associated with classic M2 or M-IL-4 macrophages, including *Chil3*, *Mrc1*, *Ccl24*, and *Il4ra*. Because IL-4 plays an important role in muscle repair^131^, it is likely that the R2 macrophages differentiated in response to environmental IL-4 or IL-13 are present in the injured muscle. The R2 gene expression profile overlapped with that of human liver macrophages^132^, suggesting that M-IL-4 macrophages might participate in tissue repair and regeneration. Further analysis of macrophages found in other tissues undergoing type II inflammatory responses will be needed to determine whether the addition of cell surface markers such as CD301b and IL-4Rα^hi^ to other “M2” marker panels, including CD206 and transglutaminase-2 (TG2)^112^, will provide consistent discrimination of macrophages activated by IL-4 or IL-13 *in vivo*. In this regard, Zhou *et al*.^133^ described an increase in CD206^+^CD103^+^ interstitial lung macrophages in response to endothelial cell–derived R-spondin 3 *in vitro* and during the repair phase of an LPS-induced lung injury model *in vivo*. The R-spondin 3-induced increase in anti-inflammatory M2-like macrophage phenotype (expressing *Mrc1*, *Arg1*, *Retnla*, *ChiL3*, and *Il10*) was necessary to limit endotoxemia-induced lung damage and death.

An enhanced understanding of the role for IL-4-activated M2 macrophages in tissue repair and host immune response will be needed to fully appreciate and take full advantage of the potential of these cells. For example, IL-4-coated gold nanoparticles were used to enhance recovery of injured skeletal muscle, resulting in a 40% increase in muscle force^134^. The IL-4 nanoparticles increased the percentage of muscle M2 macrophages twofold, and the beneficial effect was abrogated by monocyte/macrophage depletion.

A number of studies have implicated M2 macrophages as vital contributors to the anti-helminth immune response^135–137^, to the severity of allergic inflammation^119,138,139^, and to tissue repair^134,140^. However, truly understanding their function has been hampered by the lack of robust and specific mechanisms for their deletion. Methods used in the past include transiently poisoning macrophages with clodronate-loaded liposomes or conditional deletion using *LysM-Cre* deleter mice, but neither approach is specific for M2 cells^127–129^. However, a recent publication^141^ describes the generation of mice (*RetnlaCre*_R26^tdTomato^) reporting expression of RELMα, a protein induced by IL-4 in macrophages, and mice that support conditional deletion of RELMα-expressing cells by treatment with diphtheria toxin (*RetnlaCre*_R26^iDTR^). Using the reporter mice, the authors found that white adipose tissue, peritoneum, and gut were enriched with Relmα-tdTomato^+^ macrophages at a steady state. In addition, Relmα expression was detected in alveolar epithelial type 2 cells and in subsets of eosinophils and neutrophils. Interestingly, alveolar macrophages were largely negative for Relmα-dtTomato expression, even when stimulated with IL-4 *in vitro*, although a minor subset produced a low level of Relmα. These results are consistent with a study^142^ demonstrating that alveolar macrophages were much less responsive to IL-4 than peritoneal macrophages, potentially because of impaired glycolysis in the pulmonary niche. The alveolar macrophages regained responsiveness to IL-4 after their removal from the lungs in a glycolysis-dependent pathway. Surprisingly, the basal expression of Relmα-dtTomato in peritoneal macrophages was not dependent on IL-4/IL-13 or STAT6, in contrast to the low basal expression in alveolar macrophages, which was dependent on IL-4/IL-13/STAT6. Infection with the nematode *N. brasiliensis* resulted in an increase in lung Relmα-dtTomato^+^ interstitial macrophages and eosinophils in an IL-4/IL-13- and STAT6-dependent manner.

Using *RetnlaCre*_R26^iDTR^ mice, the authors showed that deletion of RELMα-expressing cells with diptheria toxin (DT) during a primary infection with *N. brasiliensis* resulted in lethality but that control mice treated with DT cleared the infection and survived. Furthermore, removal of RELMα-expressing cells by DT during a secondary infection impaired protection, as assessed by larger parasite burdens in lung and small intestine and lower parasite counts in the skin, the site of parasite entry. Similar results were obtained using a strain with a Cre-inducible DTR in CSF1R (CD115)^+^ cells (*RetnlaCre*_CD115^iDTR^) that allowed for specific deletion of RELMα^+^ macrophages. Use of these *RetnlaCre* strains, in addition to the *Csf1r*^LsL-DTR^^143^ and *CD206*^DTR^^144^ mice, will enhance the ability to specifically delete M2 macrophages and specific genes they express. This will allow the investigation of their roles in diverse effector functions, including protection from helminthic parasites, eosinophilic inflammatory diseases, tissue repair, fat and glucose metabolism, and tumor growth.

## IL-4 and chromatin remodeling in macrophages

Macrophage phenotypes are controlled by epigenetic mechanisms, including histone modifications^145–147^. Early studies demonstrated that the histone 3 Lys 27 (H3K27) demethylase jumonji-domain containing-3 (JMJD3) is essential for development of M2 cells *in vitro* and *in vivo*^148,149^. IL-4-induced activation of STAT6 resulted in increased abundance of JMJD3, which resulted in augmented expression of *Irf4* and other downstream M2 genes by regulating histone modifications^148^. JMJD3 activity resulted in methylation of H3K27, thus relieving this negative histone mark. It was further reported that IL-4 induced an increase of H3K4 methylation and acetylation of H3 bound to M2 genes, marks of active chromatin. IL-4-induced polarization to the M2 phenotype is influenced by sex differences in humans and mice^138,150^. It was recently shown that female alveolar macrophages have increased IL-4Rα and estrogen receptor (ER) α expression compared with male counterparts, and greater epigenetically poised chromatin at M2 gene promoters, while the promoters were epigenetically silenced in male macrophages^151^. Furthermore, estradiol (E2) acting through ERα supported enhanced expression of IL-4-induced M2 genes. Exposure of female macrophages to E2 permanently changed the responsiveness of the cells to subsequent Th2 inflammatory stimulus even after macrophages were removed from all exogenous E2 up to 10 days later. These data have important implications for diseases in which E2 and macrophages play a key role, such as allergic asthma and breast cancer, and suggest that sex may influence the outcome of therapeutics targeting macrophages^151^.

Large-scale “omic” studies have recently shown that opposing macrophage polarization programs induced by IL-4 versus IFNγ show epigenomic and transcriptional cross-regulation^152^, and each cytokine is able to suppress a subset of genes regulated by the opposing cytokine. Upon IL-4 stimulation of macrophages, greater H3K27 acetylation was observed in genomic regions with high representation of canonical binding sites for STAT6. These regions were mainly near IL-4-inducible genes that were suppressed by IFNγ. Interestingly, a small set of IL-4-regulated genomic regions resistant to IFNγ suppression was enriched for Myc-binding E-box sequences. IL-4 increased the abundance of Myc protein and this was important for the induction of genes resistant to IFNγ suppression, including *Ccl2*, *Ccl17*, and *Ccl12*^152^. Tang *et al*. further showed that IL-4-induced changes in chromatin accessibility can differ in different types of macrophages^153^. The regions of chromatin altered by IL-4 were enriched for PU.1 binding motifs, notably in intronic regions capable of modulating higher-order structure of DNA (minor groove width and twist). Polymorphisms in C57BL/6 versus BALB/c mice in sequences flanking PU.1 binding motifs that altered DNA shape configurations were shown to contribute to differences in chromatin accessibility in response to IL-4. Moreover, global transcriptional profiling showed that macrophages from the different strains responded differently to IL-4. For example, IL-4-stimulated macrophages from BALB/c mice expressed lower levels of cell cycle–related genes, PDL-2, and MHC class II than macrophages from C57BL/6 mice. These results support the model that genetic polymorphisms that cause alterations in local DNA structure can influence changes in chromatin accessibility stimulated by IL-4. Whether this is directly correlated with differences in their transcriptional responses to IL-4 remains to be determined^153^. Furthermore, it has not yet been determined whether modulating epigenetic pathways will be useful in controlling macrophage polarization in disease states^154^. Indeed, in addition to positive effects, STAT6 has been shown to repress a set of inflammatory enhancers, thereby limiting the activation of AAMs^155^. Moreover, IL-4 via STAT6 can promote TET2-dependent demethylation to favor DC differentiation^156^; conversely, DCs can act to reinforce Th2 cytokine production in allergic disease^157^.

## IL-4 and neutrophil function

Neutrophils are normally considered the first line of defense against bacterial infections and are the first immune cell type to enter a site of inflammation. Neutrophilic infiltration is most associated with Th1 and Th17 immune responses, but neutrophils also have been reported to play important roles in anti-helminth immunity^158–160^. Priming of macrophages to become M2 cells and engage in the killing and clearance of helminthic parasites was dependent on the presence of neutrophils^159^. In addition, both mouse and human neutrophils can kill helminth larvae *in vitro* with the cooperation of macrophages^158^, and neutrophils and macrophages have been found clustered around parasites *in vivo*^160^, suggesting that neutrophils play a cooperative role in type II immune responses. On the other hand, recent reports demonstrate that IL-4 signaling suppresses neutrophil function. Woytschak *et al*. reported that IL-4 treatment of neutrophils inhibited their migration toward CXCL2 and reduced expression of CXCR2^161^. Furthermore, treatment of mice infected with the bacteria led to reduced neutrophil counts, reduced neutrophil migration, increased bacterial burden, and decreased survival. Neutrophils isolated from IL-4-treated mice had reduced expression of CXCR2 and elevated expression of CXCR4. Similarly, Impellizzieri *et al*. showed that treatment of human neutrophils with IL-4 inhibited their migration to CXCL2 and reduced the formation of extracellular neutrophil extracellular traps^162^. Thus, it has been hypothesized that neutrophils assist macrophages early in the course of a nematode infection, but as Th2 cells arrive and produce high levels of IL-4, neutrophil function is downregulated to limit neutrophil-mediated tissue damage^163^. Future studies will be needed to determine whether these pathways can be targeted in various infectious or inflammatory diseases.

## Therapeutic manipulation of the actions of IL-4

Agents that either increase or inhibit IL-4 activity have been generated. Although it is less clear from a therapeutic perspective that augmenting IL-4 would be beneficial, engineered IL-4 “superkines” have been generated. A type I IL-4 receptor–selective IL-4 superkine exhibits marked enhanced binding for γ_c_ and exhibits 3- to 10-fold enhanced potency for STAT6 activation, whereas an IL-4 variant with enhanced affinity for IL-13Rα1 more potently drives the differentiation of monocyte-derived DCs^164^.

One of the most exciting molecules from a therapeutic perspective is a fully humanized IgG4 monoclonal antibody, dupilumab, to IL-4Rα that binds to an epitope on the IL-4Rα chain required for receptor dimer formation and thereby inhibits the actions of both IL-4 and IL-13. Dupilumab was approved by the US Food and Drug Administration in 2017 for the treatment of moderate to severe atopic dermatitis and subsequently for add-on treatment of moderate to severe asthma with eosinophilic phenotype in patients age 12 or older^165,166^, and there was an 87% decrease in asthma exacerbation in one study^167^. Another monoclonal antibody, lebrikizumab, which blocks the actions of IL-13 but not IL-4, has been shown to be effective in treating asthma^168^. Pitrakinra is a variant of human IL-4 with mutations in helix D that are critical for dimerization of IL-4Rα formation with γ_c_ or IL-13Rα1 and thus blocks signaling by IL-4 or IL-13. Pitrakinra has shown some efficacy in the treatment of asthma in a subset of patients^169^. Tralokinumab is another IL-13-based inhibitor. Thus, monoclonal antibodies, engineered cytokines, and potentially small molecules are ways to fine-tune the signal strength of IL-4 or IL-13 (or both) and represent next-generation approaches for treating allergic and inflammatory diseases. It will be interesting to assess the potency and relative utility of agents that selectively target type I versus type II immunity versus the combination of both type I and type II immunity.

Besides anti-IL-4Rα-based therapies, reagents targeting the Fc region of IgE (omalizumab), the α subunit of IL-5 (reslizumab, mepolizumab—phase 2b studies), domain 1 of IL-5Rα (benralizumab), and TSLP (tezepelumab-phase 2a study) are under active investigation^170,171^.

Finally, because type I IL-4 receptors contain γ_c_ as a critical component and IL-4 activates JAK1 and JAK3 via these receptors, one must be cognizant of the inhibition of IL-4 signaling by JAK inhibitors (Jakinibs) that target either of these JAK kinases. Indeed, a number of Jakinibs are being evaluated for atopic dermatitis, with topical delgocitinib having recently been approved in Japan for the treatment of adults with this disease and other agents under ongoing evaluation^172^.

## Conclusions

In a little under 40 years, IL-4 has expanded from being discovered as a factor with biological actions on B cells to a molecularly defined molecule with diverse actions on a range of cell lineages. Accordingly, manipulating the actions of IL-4 has broad ramifications, including related to allergy, cancer, and even the central nervous system. Besides the enormous new basic science information that has accrued, the therapeutic manipulation of the actions of this cytokine has already shown promise in disease states. Better understanding its actions and signaling mechanisms will continue to provide new information and targets to further manipulate its actions.
